# Bis(acetato-κ^2^ *O*,*O*′)bis­[4-(dimethyl­amino)­pyridine-κ*N*]copper(II)

**DOI:** 10.1107/S1600536811002017

**Published:** 2011-01-22

**Authors:** Meriem Benslimane, Hocine Merazig, Jean-Claude Daran

**Affiliations:** a Unité de Recherche de Chimie de l’Environnement et Moléculaire Structurale, Faculté des Sciences Exactes, Département de Chimie, Université Mentouri de Constantine, 25000 Constantine, Algeria; b Laboratoire de Chimie de Coordination, UPR-CNRS 8241, 05 route de Narbonne, 31077 Toulouse Cedex 4, France

## Abstract

In the mononuclear title complex, [Cu(CH_3_COO)_2_(C_7_H_10_N_2_)_2_], the Cu^II^ ion, located on a crystallographic inversion centre, is six coordinated by two N atoms of two 4-(dimethyl­amino)­pyridine (DMAP) ligands in apical positions and four O atoms from two symmetry-related opposite acetate anions, which are asymmetrically bonded in the equatorial plane. The complex and the crystal packing of the complex are stabilized by intra- and inter­molecular C—H⋯O hydrogen bonds, giving *R*_4_^2^(10) rings and generating a layer-like structure.

## Related literature

For the importance of copper(II) carboxyl­ate complexes in biology, see: Lippard & Berg (1994 ▶). For coordination properties of carboxyl­ates, see: Deacon & Phillips (1980 ▶). For a similar structure, see: Li *et al.* (2009 ▶). For bond lengths in related copper complexes, see: Cui *et al.* (2009 ▶); Zaleski *et al.* (2005 ▶). For graph-set motifs, see: Bernstein *et al.* (1995 ▶).
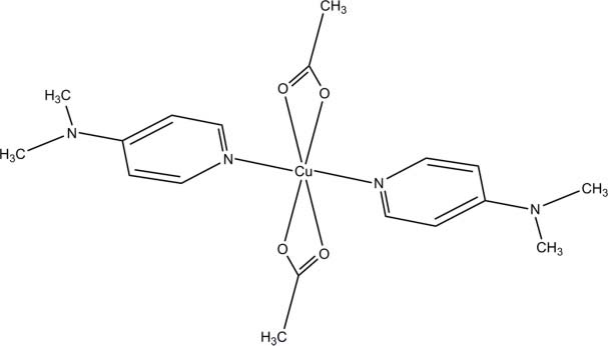


## Experimental

### 

#### Crystal data

[Cu(C_2_H_3_O_2_)_2_(C_7_H_10_N_2_)_2_]
*M* *_r_* = 425.98
Triclinic, 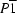

*a* = 7.6930 (2) Å
*b* = 7.8331 (2) Å
*c* = 8.2206 (2) Å
α = 90.701 (2)°
β = 96.992 (2)°
γ = 92.949 (2)°
*V* = 490.95 (2) Å^3^
*Z* = 1
Mo *K*α radiation
μ = 1.14 mm^−1^
*T* = 180 K
0.48 × 0.37 × 0.12 mm

#### Data collection

Agilent Xcalibur Eos Gemini-ultra diffractometer
Absorption correction: multi-scan [ABSPACK in *CrysAlis PRO* (Agilent Technologies, 2010 ▶)] *T*_min_ = 0.608, *T*_max_ = 0.872
10140 measured reflections
2362 independent reflections
2307 reflections with *I* > 2σ(*I*)
*R*_int_ = 0.018

#### Refinement

*R*[*F* ^2^ > 2σ(*F* ^2^)] = 0.027
*wR*(*F* ^2^) = 0.115
*S* = 1.11
2362 reflections
124 parameters
H-atom parameters constrained
Δρ_max_ = 0.40 e Å^−3^
Δρ_min_ = −0.36 e Å^−3^

### 

Data collection: *CrysAlis PRO* (Agilent Technologies, 2010 ▶); cell refinement: *CrysAlis PRO*; data reduction: *CrysAlis PRO*; program(s) used to solve structure: *SIR92* (Altomare *et al.*, 1994 ▶); program(s) used to refine structure: *CRYSTALS* (Betteridge *et al.*, 2003 ▶); molecular graphics: *ORTEP-3 for Windows* (Farrugia, 1997 ▶); software used to prepare material for publication: *CRYSTALS*.

## Supplementary Material

Crystal structure: contains datablocks global, I. DOI: 10.1107/S1600536811002017/su2247sup1.cif

Structure factors: contains datablocks I. DOI: 10.1107/S1600536811002017/su2247Isup2.hkl

Additional supplementary materials:  crystallographic information; 3D view; checkCIF report

## Figures and Tables

**Table 1:** Hydrogen-bond geometry (Å, °)

*D*—H⋯*A*	*D*—H	H⋯*A*	*D*⋯*A*	*D*—H⋯*A*
C8—H81⋯O4^i^	0.95	2.51	3.452 (2)	173
C10—H101⋯O2^ii^	0.93	2.49	3.381 (2)	161

Symmetry codes: (i) 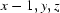; (ii) 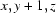.

**Fig. 1.**
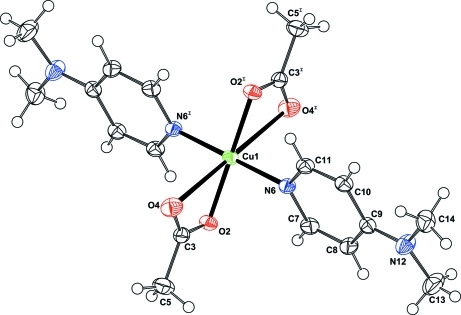
The molecular structure of the title compound with displacement ellipsoids drawn at the 30% probability level. H atoms are shown as spheres of arbitary radius [Symmetry code: (I) = -x + 1,-y + 1,-z + 1].

**Fig. 2.**
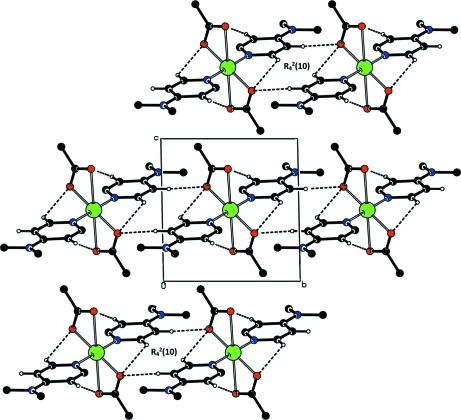
A view along the a-axis of the crystal structure of the title compound showing the formation of R42(10) rings. The C-H···O hydrogen bonds are shown as dashed lines; H-atoms no involved in the C-H..O interactions have been omitted for clarity.

## supplementary crystallographic information

### Comment

Lewis based coordinated Cu^II^ carboxylate complexes are an important class of coordination compounds due to their relevance as structural and functional models for biologically important metalloenzymes (Lippard & Berg,1994). Anionic carboxylates are highly flexible and versatile O-donor ligands since a range of substituents may be introduced on the alkyl chain to modulate their reactivity and coordination propensity, and result in a variety of coordination modes such as monodentate, bidentate bridging, chelating, monoatomic bridging and chelating bridging (Deacon & Phillips, 1980). The Lewis base 4-Dimethylaminopyridine (DMAP) is a derivative of pyridine that is widely used in hypernucleophilic acylation for a variety of reactions, such as esterifications with anhydrides. We report herein on the molecular structure of a novel compound, namely bis(acetate-*κ^2^O,O'*)bis(4-dimethylaminepyridine-*κN*)] Copper(II).

In the title complex the Cu^II^ cation lies on an inversion centre, as a consequence of which the asymmetric unit comprises one half-molecule (Fig. 1). The Cu^II^ ion is octahedrally coordinated by two (DMAP) ligands and two acetate units. It adopts a Jahn-Teller-distorted *trans*-CuO_4_N_2_ octahedral coordination similar to our previously reported Cu^II^ compound with the 4-(pyridine-4-yl)pyrimidine-2-sulfonate ligand (Li *et al.*, 2009). The four O atoms [O2, O4, and the symmetry-related atoms, O2^I^, O4^I^ (symmetry code: (I) -*x* + 1,-*y* + 1,-*z* + 1)] are located in the equatorial plane while the two N atoms of the (DMAP) ligands (N6, N6^I^) are in the axial positions. The Cu1—N6 bond length of 2.0095 (13) Å agrees well with that reported for related copper complexes (Cui *et al.*, 2009, Zaleski *et al.*, 2005), while the Cu1—O2 and Cu1—O4 bond lengths are 1.9715 (11) and 2.5932 (13) Å, respectively. The dihedral angles formed between the mean planes through the four O atoms and the pyridine ring is 88.59 (1)°.

In the crystal, the packing is consolidated by C—H···O interactions involving aromatic H-atoms (Table 1, Fig 2), in which *R*_4_^2^(10) (Bernstein *et al.*,1995) hydrogen-bonded rings are formed, generating a two-dimensional layer-like structure.

### Experimental

To a solution of Cu(CH_3_CO_2_)_2_.H_2_O (0.2 g, 1 mmol) in methanol (40 cm^3^) at room temperature was added solid 4-(Dimethylamino)pyridine (DMAP) (0.122 g, 1 mmol) in small portions under constant stirring. the mixture was then filtered and the filtrate allowed to stand for 20 days, after which small blue block-like crystals of the title complex were obtained. They were filtered and dried under vacuum.

### Refinement

All the C-bound H-atoms were located in difference Fourier maps but were treated as riding on their parent atoms: C-H = 0.917 - 0.974 Å with *U*_iso_(H) = 1.2*U*_eq_(C-aromatic) or *U*_iso_(H) = 1.5*U*_eq_(C-methyl).

### Crystal data

**Table:**

[Cu(C_2_H_3_O_2_)_2_(C_7_H_10_N_2_)_2_]	*Z* = 1
*M**_r_* = 425.98	*F*(000) = 223
Triclinic, *P*1	*D*_x_ = 1.441 Mg m^−^^3^
Hall symbol: -P 1	Mo *K*α radiation, λ = 0.71073 Å
*a* = 7.6930 (2) Å	Cell parameters from 10054 reflections
*b* = 7.8331 (2) Å	θ = 3.4–29.0°
*c* = 8.2206 (2) Å	µ = 1.14 mm^−^^1^
α = 90.701 (2)°	*T* = 180 K
β = 96.992 (2)°	Plate, blue
γ = 92.949 (2)°	0.48 × 0.37 × 0.12 mm
*V* = 490.95 (2) Å^3^	

### Data collection

**Table:**

Agilent Xcalibur Eos Gemini-ultra diffractometer	2362 independent reflections
Radiation source: Enhance (Mo) X-ray Source	2307 reflections with *I* > 2σ(*I*)
graphite	*R*_int_ = 0.018
Detector resolution: 16.1978 pixels mm^-1^	θ_max_ = 29.1°, θ_min_ = 3.4°
ω scans	*h* = −10→10
Absorption correction: multi-scan [ABSPACK in *CrysAlis PRO* (Agilent Technologies, 2010)]	*k* = −10→10
*T*_min_ = 0.608, *T*_max_ = 0.872	*l* = −11→11
10140 measured reflections	

### Refinement

**Table:**

Refinement on *F*^2^	Primary atom site location: structure-invariant direct methods
Least-squares matrix: full	Hydrogen site location: inferred from neighbouring sites
*R*[*F*^2^ > 2σ(*F*^2^)] = 0.027	H-atom parameters constrained
*wR*(*F*^2^) = 0.115	Method = Modified Sheldrick *w* = 1/[σ^2^(*F*^2^) + (0.1*P*)^2^ + 0.0*P*], where *P* = p(6)*max(*F*_o_^2^,0) + (1-p(6))*F*_c_^2^
*S* = 1.11	(Δ/σ)_max_ = 0.001
2362 reflections	Δρ_max_ = 0.40 e Å^−^^3^
124 parameters	Δρ_min_ = −0.36 e Å^−^^3^
0 restraints	

### Fractional atomic coordinates and isotropic or equivalent isotropic displacement parameters (Å^2^)

**Table:**

	*x*	*y*	*z*	*U*_iso_*/*U*_eq_	
Cu1	0.5000	0.5000	0.5000	0.0192	
O2	0.44954 (15)	0.32890 (15)	0.66419 (14)	0.0229	
C3	0.5609 (2)	0.3691 (2)	0.79019 (19)	0.0222	
O4	0.67659 (17)	0.48560 (17)	0.78860 (16)	0.0321	
C5	0.5412 (3)	0.2708 (3)	0.9440 (2)	0.0328	
N6	0.32934 (17)	0.65325 (17)	0.58644 (16)	0.0204	
C7	0.1803 (2)	0.5914 (2)	0.6416 (2)	0.0244	
C8	0.0597 (2)	0.6918 (2)	0.6986 (2)	0.0247	
C9	0.0888 (2)	0.8714 (2)	0.70624 (18)	0.0217	
C10	0.2452 (2)	0.9354 (2)	0.64866 (19)	0.0226	
C11	0.3565 (2)	0.8244 (2)	0.59137 (19)	0.0228	
N12	−0.0240 (2)	0.9759 (2)	0.7651 (2)	0.0320	
C13	−0.1935 (3)	0.9125 (3)	0.8063 (3)	0.0419	
C14	0.0055 (3)	1.1609 (2)	0.7637 (2)	0.0338	
H51	0.6487	0.2899	1.0188	0.0450*	
H53	0.4473	0.3138	0.9949	0.0447*	
H52	0.5228	0.1509	0.9233	0.0442*	
H71	0.1609	0.4749	0.6399	0.0287*	
H81	−0.0432	0.6385	0.7331	0.0283*	
H101	0.2738	1.0521	0.6478	0.0252*	
H111	0.4575	0.8681	0.5535	0.0258*	
H132	−0.2467	0.9992	0.8633	0.0610*	
H131	−0.1792	0.8177	0.8754	0.0606*	
H133	−0.2691	0.8797	0.7128	0.0611*	
H142	−0.0664	1.2144	0.8370	0.0514*	
H141	0.1245	1.1930	0.8012	0.0513*	
H143	−0.0217	1.1993	0.6517	0.0513*	

### Atomic displacement parameters (Å^2^)

**Table:**

	*U*^11^	*U*^22^	*U*^33^	*U*^12^	*U*^13^	*U*^23^
Cu1	0.02144 (19)	0.01507 (18)	0.02130 (19)	−0.00171 (11)	0.00403 (11)	0.00410 (11)
O2	0.0263 (6)	0.0200 (5)	0.0222 (5)	−0.0024 (4)	0.0037 (4)	0.0049 (4)
C3	0.0243 (7)	0.0201 (7)	0.0236 (7)	0.0039 (6)	0.0072 (5)	0.0023 (6)
O4	0.0290 (6)	0.0317 (7)	0.0348 (7)	−0.0083 (5)	0.0055 (5)	−0.0006 (5)
C5	0.0434 (10)	0.0332 (10)	0.0233 (8)	0.0038 (8)	0.0085 (7)	0.0068 (7)
N6	0.0203 (6)	0.0167 (6)	0.0245 (6)	−0.0022 (5)	0.0047 (5)	0.0027 (5)
C7	0.0250 (8)	0.0176 (7)	0.0305 (8)	−0.0049 (6)	0.0043 (6)	0.0045 (6)
C8	0.0206 (7)	0.0203 (7)	0.0331 (8)	−0.0051 (5)	0.0053 (6)	0.0035 (6)
C9	0.0213 (7)	0.0203 (7)	0.0226 (7)	−0.0017 (5)	0.0001 (5)	0.0024 (6)
C10	0.0243 (7)	0.0170 (7)	0.0258 (7)	−0.0040 (5)	0.0030 (6)	0.0019 (6)
C11	0.0225 (7)	0.0196 (8)	0.0259 (8)	−0.0049 (6)	0.0035 (6)	0.0037 (6)
N12	0.0270 (7)	0.0237 (7)	0.0470 (9)	−0.0012 (6)	0.0119 (6)	0.0003 (7)
C13	0.0266 (9)	0.0430 (11)	0.0583 (12)	−0.0011 (8)	0.0158 (8)	0.0032 (9)
C14	0.0341 (9)	0.0229 (8)	0.0446 (10)	0.0044 (7)	0.0050 (7)	0.0000 (7)

### Geometric parameters (Å, °)

**Table:**

Cu1—O2	1.9715 (11)	C7—H71	0.917
Cu1—C3	2.6076 (16)	C8—C9	1.413 (2)
Cu1—O4	2.5932 (13)	C8—H81	0.951
Cu1—N6	2.0095 (13)	C9—C10	1.416 (2)
Cu1—O4^i^	2.5932 (13)	C9—N12	1.350 (2)
Cu1—C3^i^	2.6076 (16)	C10—C11	1.370 (2)
Cu1—N6^i^	2.0095 (13)	C10—H101	0.929
Cu1—O2^i^	1.9715 (11)	C11—H111	0.923
O2—C3	1.286 (2)	N12—C13	1.451 (2)
C3—O4	1.243 (2)	N12—C14	1.455 (2)
C3—C5	1.507 (2)	C13—H132	0.958
C5—H51	0.972	C13—H131	0.943
C5—H53	0.951	C13—H133	0.931
C5—H52	0.953	C14—H142	0.972
N6—C7	1.353 (2)	C14—H141	0.950
N6—C11	1.3452 (19)	C14—H143	0.974
C7—C8	1.367 (2)		
			
O4^i^—Cu1—C3^i^	27.66 (5)	H51—C5—H53	108.3
O4^i^—Cu1—N6^i^	91.06 (5)	C3—C5—H52	112.4
C3^i^—Cu1—N6^i^	89.28 (5)	H51—C5—H52	108.0
O4^i^—Cu1—O2^i^	56.16 (4)	H53—C5—H52	110.7
C3^i^—Cu1—O2^i^	28.54 (5)	Cu1—N6—C7	122.31 (11)
N6^i^—Cu1—O2^i^	89.50 (5)	Cu1—N6—C11	121.62 (10)
O4^i^—Cu1—O2	123.84 (4)	C7—N6—C11	116.07 (13)
C3^i^—Cu1—O2	151.46 (5)	N6—C7—C8	123.95 (14)
N6^i^—Cu1—O2	90.50 (5)	N6—C7—H71	116.8
O2^i^—Cu1—O2	179.994	C8—C7—H71	119.2
O4^i^—Cu1—C3	152.34 (5)	C7—C8—C9	120.21 (14)
C3^i^—Cu1—C3	179.996	C7—C8—H81	118.9
N6^i^—Cu1—C3	90.72 (5)	C9—C8—H81	120.9
O2^i^—Cu1—C3	151.46 (5)	C8—C9—C10	115.59 (14)
O2—Cu1—C3	28.54 (5)	C8—C9—N12	122.54 (14)
O4^i^—Cu1—O4	179.996	C10—C9—N12	121.87 (14)
C3^i^—Cu1—O4	152.34 (5)	C9—C10—C11	119.82 (14)
N6^i^—Cu1—O4	88.94 (5)	C9—C10—H101	121.3
O2^i^—Cu1—O4	123.84 (4)	C11—C10—H101	118.9
O2—Cu1—O4	56.16 (4)	C10—C11—N6	124.35 (14)
O4^i^—Cu1—N6	88.94 (5)	C10—C11—H111	118.8
C3^i^—Cu1—N6	90.72 (5)	N6—C11—H111	116.8
N6^i^—Cu1—N6	179.994	C9—N12—C13	121.83 (16)
O2^i^—Cu1—N6	90.50 (5)	C9—N12—C14	121.12 (15)
O2—Cu1—N6	89.50 (5)	C13—N12—C14	116.23 (16)
C3—Cu1—O4	27.66 (5)	N12—C13—H132	110.3
C3—Cu1—N6	89.28 (5)	N12—C13—H131	109.8
O4—Cu1—N6	91.06 (5)	H132—C13—H131	108.1
Cu1—O2—C3	104.37 (9)	N12—C13—H133	111.4
Cu1—C3—O2	47.09 (7)	H132—C13—H133	108.3
Cu1—C3—O4	75.53 (10)	H131—C13—H133	109.0
O2—C3—O4	122.50 (15)	N12—C14—H142	110.0
Cu1—C3—C5	162.83 (12)	N12—C14—H141	110.5
O2—C3—C5	116.55 (14)	H142—C14—H141	107.5
O4—C3—C5	120.92 (15)	N12—C14—H143	108.7
Cu1—O4—C3	76.82 (9)	H142—C14—H143	111.3
C3—C5—H51	108.2	H141—C14—H143	108.8
C3—C5—H53	109.2		

Symmetry codes: (i) −*x*+1, −*y*+1, −*z*+1.

### Hydrogen-bond geometry (Å, °)

**Table:**

*D*—H···*A*	*D*—H	H···*A*	*D*···*A*	*D*—H···*A*
C8—H81···O4^ii^	0.95	2.51	3.452 (2)	173
C10—H101···O2^iii^	0.93	2.49	3.381 (2)	161
C11—H111···O2^i^	0.92	2.54	2.9946 (19)	111

Symmetry codes: (ii) *x*−1, *y*, *z*; (iii) *x*, *y*+1, *z*; (i) −*x*+1, −*y*+1, −*z*+1.
